# Point of Care Ultrasound in Monitoring of Post-Renal Biopsy Bleeding 

**DOI:** 10.24908/pocus.v7iKidney.14988

**Published:** 2022-02-01

**Authors:** Natalie N McCall, Anna Burgner

**Affiliations:** 1 Division of Nephrology, Vanderbilt University Medical Center Nashville, TN

**Keywords:** POCUS, Renal ultrasound, Renal biopsy

## Abstract

A 32-year-old male presented with hypertensive emergency and features of thrombotic microangiopathy. He underwent a kidney biopsy after renal dysfunction persisted despite clinical improvement otherwise. The kidney biopsy was performed with direct ultrasound guidance. The procedure was complicated by hematoma formation and persistent turbulent flow on color Doppler concerning for ongoing bleeding. Serial point of care ultrasounds of the kidney with color flow Doppler were used to monitor the size of the hematoma and determine if there was evidence of ongoing bleeding. These serial ultrasounds showed stable hematoma size, resolution of biopsy-associated Doppler signal and prevented further invasive interventions.

## Case Report

A 32-year-old male with type 1 diabetes mellitus presented to the emergency department with altered mental status, hypertensive emergency, microangiopathic hemolytic anemia (MAHA), and acute kidney injury (AKI). Brain imaging showed diffuse edema of the pons and medullary pyramids concerning for atypical posterior reversible encephalopathy syndrome (PRES). He was admitted to the ICU for hypertension control and further monitoring. He was presumed to have a thrombotic microangiopathy (TMA) in the setting of malignant hypertension, but despite improvements in his mental status and stabilization of his AKI with intensive blood pressure control he had evidence of persistent MAHA with ongoing schistocytosis. A kidney biopsy was pursued to determine if additional tissue characteristics would identify alternative cause of his presumed TMA. 

**Figure 1:**
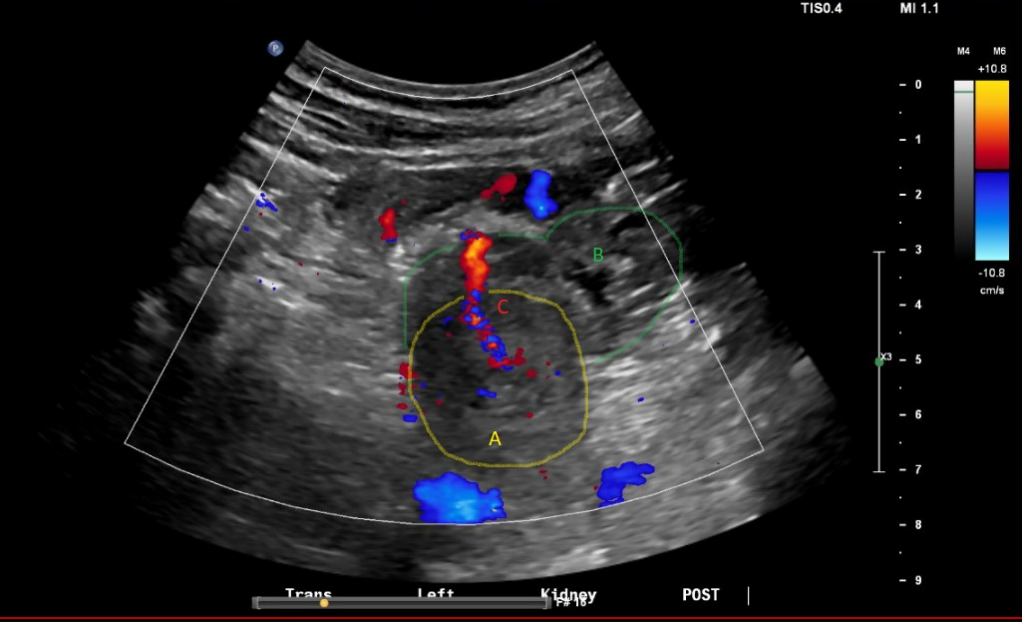
(A) Left kidney, lower pole; (B) hematoma; (C) Doppler flow signal from vascular jet. Initial ultrasound showing persistent bleeding evidenced by ongoing tract of turbulent flow on color Doppler. Hematoma size 7.8 x 1.8 x 4.7cm.

**Figure 2:**
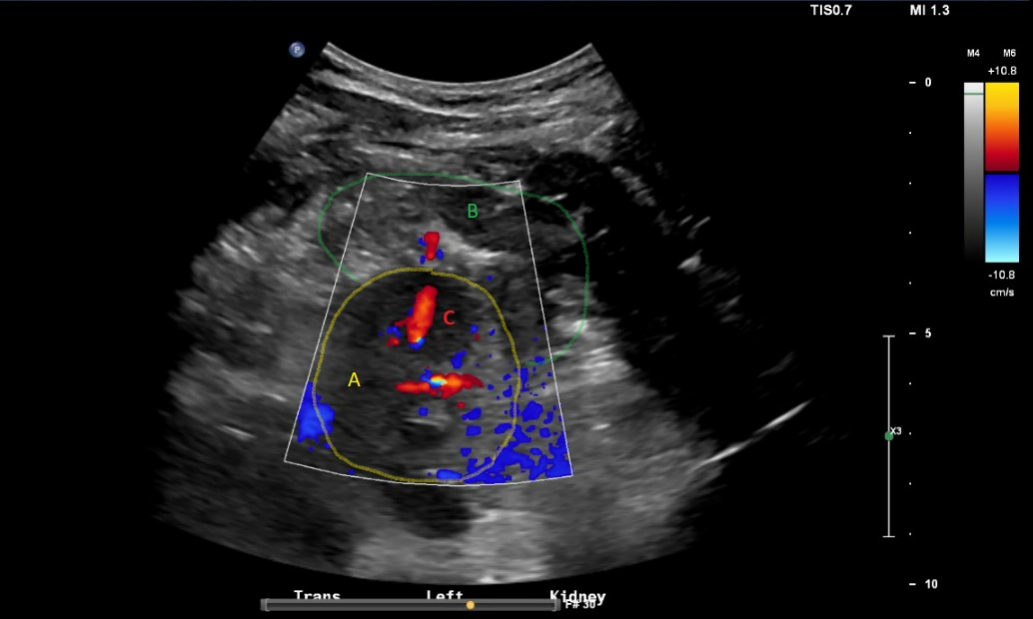
(A) Left kidney, lower pole; (B) hematoma; (C) Doppler flow signal from vascular jet. Ultrasound at 90 minutes after biopsy. Persistent bleeding evidenced by ongoing tract of turbulent flow on color Doppler. Hematoma size 7.9 x 2.3 x 4.9cm.

A left kidney biopsy was performed using a 16-gauge biopsy needle with direct ultrasound guidance. The biopsy was performed by clinical nephrology faculty and nephrology fellow. The biopsy was performed in the patient’s hospital floor room in a unit designated for renal biopsies. Three passes were performed. After the third pass, a post-procedural hematoma and tract of turbulent flow on color Doppler was noted at the lower pole of the left kidney concerning for ongoing bleeding (Figure 1). Pressure was held and the site was monitored for 30 minutes. Between 15 minutes and 30 minutes post-procedure, the size of the hematoma was unchanged and the flow on color Doppler decreased. He remained normotensive and pain was minimal. Given the stable hematoma size on ultrasound and reassuring clinical parameters, a plan for serial ultrasounds throughout the afternoon was made. Repeat renal ultrasound was performed 90 minutes after the procedure which showed persistent tract of flow on Doppler but similar hematoma size. The Doppler signal was also less intense (Figure 2). A third ultrasound, performed 4 hours after initial procedure, demonstrated stable hematoma size and no further evidence of Doppler signal suggesting no ongoing active extravasation. The follow-up ultrasounds were performed by a radiology ultrasound technician with the nephrology attending in observation and involved in clinical decision making in real time. Referral ultrasound images were additionally read by a radiologist. 

He was monitored closely overnight as per standard hospital post-kidney biopsy protocols. His hematocrit was checked every 4 hours and remained stable. Results of the biopsy indicated severe diabetic nephropathy, accelerated hypertension related injury but no evidence of TMA. He was discharged the following day. At his clinic follow-up 2 weeks after discharge, his hematocrit was stable and he had no further evidence of complications from kidney biopsy. 

The most common complication of kidney biopsies is bleeding 1, 2. Hematomas are reported in up to 57% to 91% of native kidney biopsies by CT and have been reported up to 86% of cases by ultrasound 1, 3. Most commonly, hematomas are clinically silent and do not require additional intervention 4,5 . In patients who do have clinically significant complications, hematomas are noted by 1 hour post biopsy in 77% of cases and were measured at >3cm in greater than 50% of cases 3. Presence of a color tract on doppler has also been shown to be 10.7 times more likely to result in major and minor complications 6. 

Major bleeding complications include need for transfusion, need for secondary procedures to stop arterial bleeding, and death. These complications occur in up to 2% of native kidney biopsies with 1.6% (1 in 625) requiring transfusion, 0.3% (1 in 333) requiring secondary intervention to stop arterial bleeding after biopsy, and 0.008% (1 in 12,500) resulting in death 2. At our institution, our first line therapy for secondary intervention is an arteriogram with dedicated coil and or embolization. 

In this case, the size of the hematoma and velocity of the Doppler signal at the end of the procedure were concerning and suggested that the patient may require a secondary intervention to halt the bleeding. POCUS allowed us to monitor hematoma size and the velocity of the Doppler signal of the bleeding in real time. Serial monitoring with POCUS enabled demonstrable cessation of bleeding, stability of the hematoma in size, and deferral of any emergent secondary intervention with angiogram or embolization. 

## Disclosures

None
